# Supplementary figures and images for: Maxicircle architecture and evolutionary insights into Trypanosoma cruzi complex
Source: PLoS Negl Trop Dis. 2021 Aug 26;15(8):e0009719. doi: 10.1371/journal.pntd.0009719 (PMC8425572; doi:10.1371/journal.pntd.0009719)

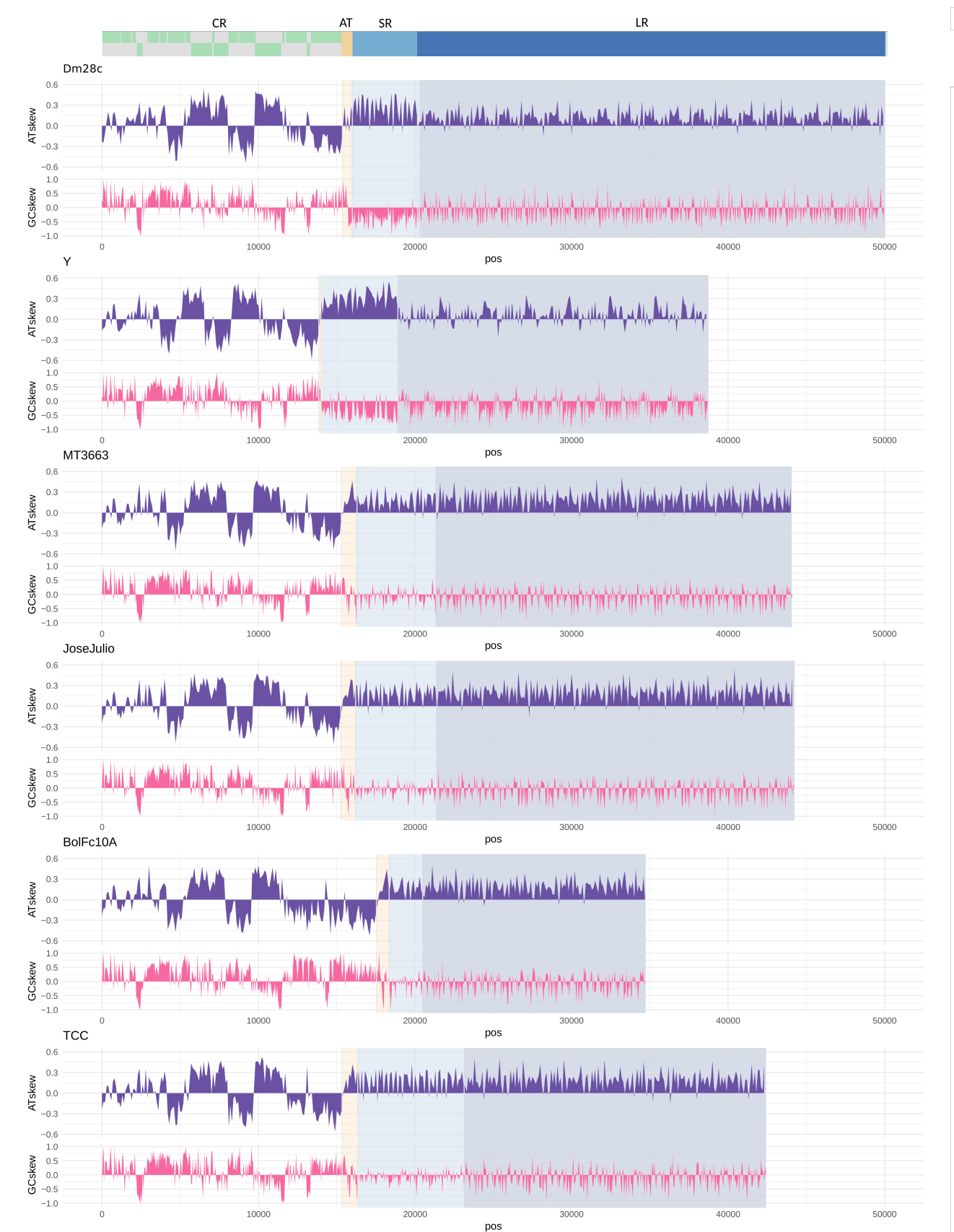

Supplement: S2 Fig — (TIF) [file pntd.0009719.s002.tif]

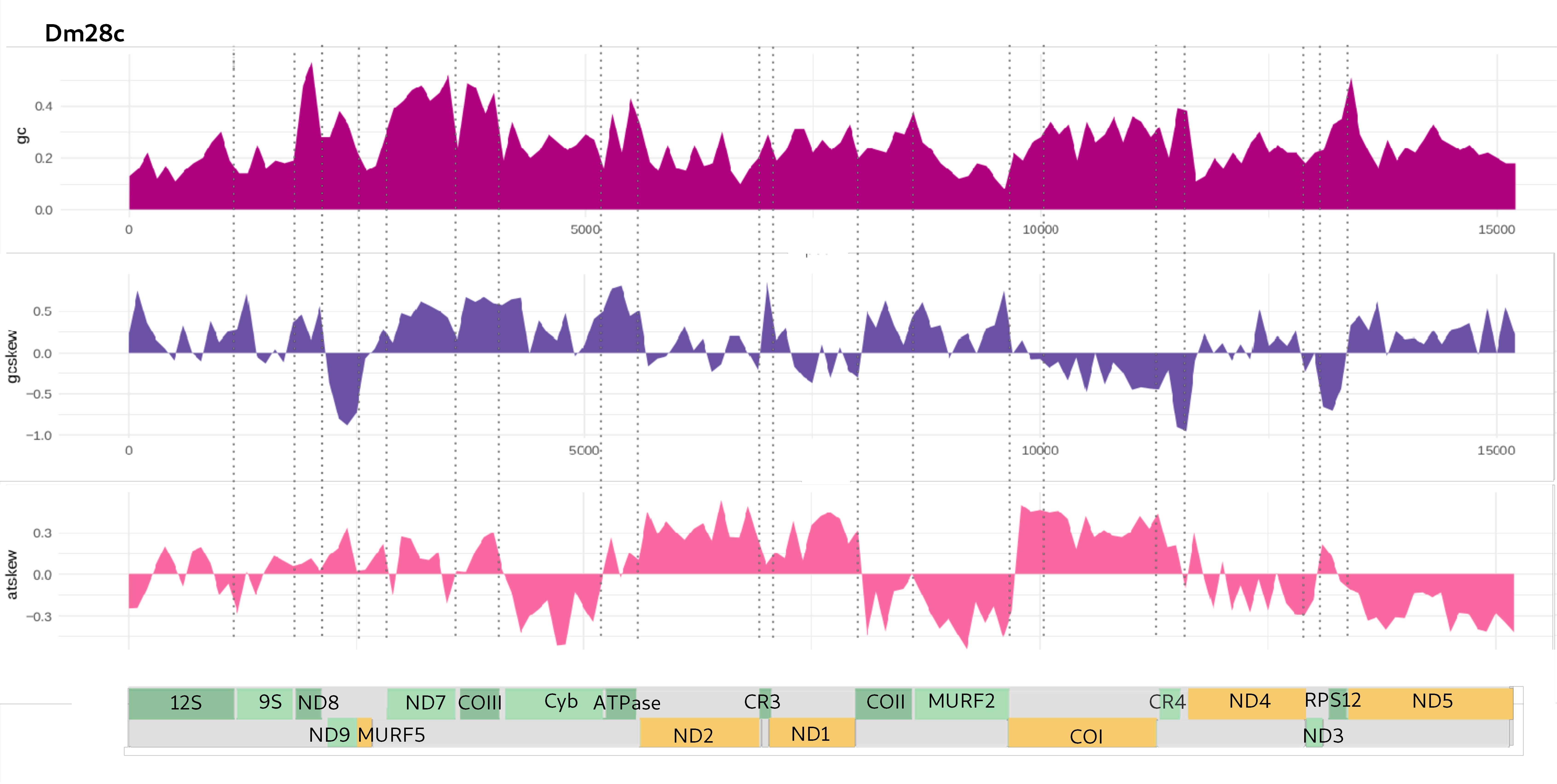

Supplement: S3 Fig — (TIF) [file pntd.0009719.s003.tif]

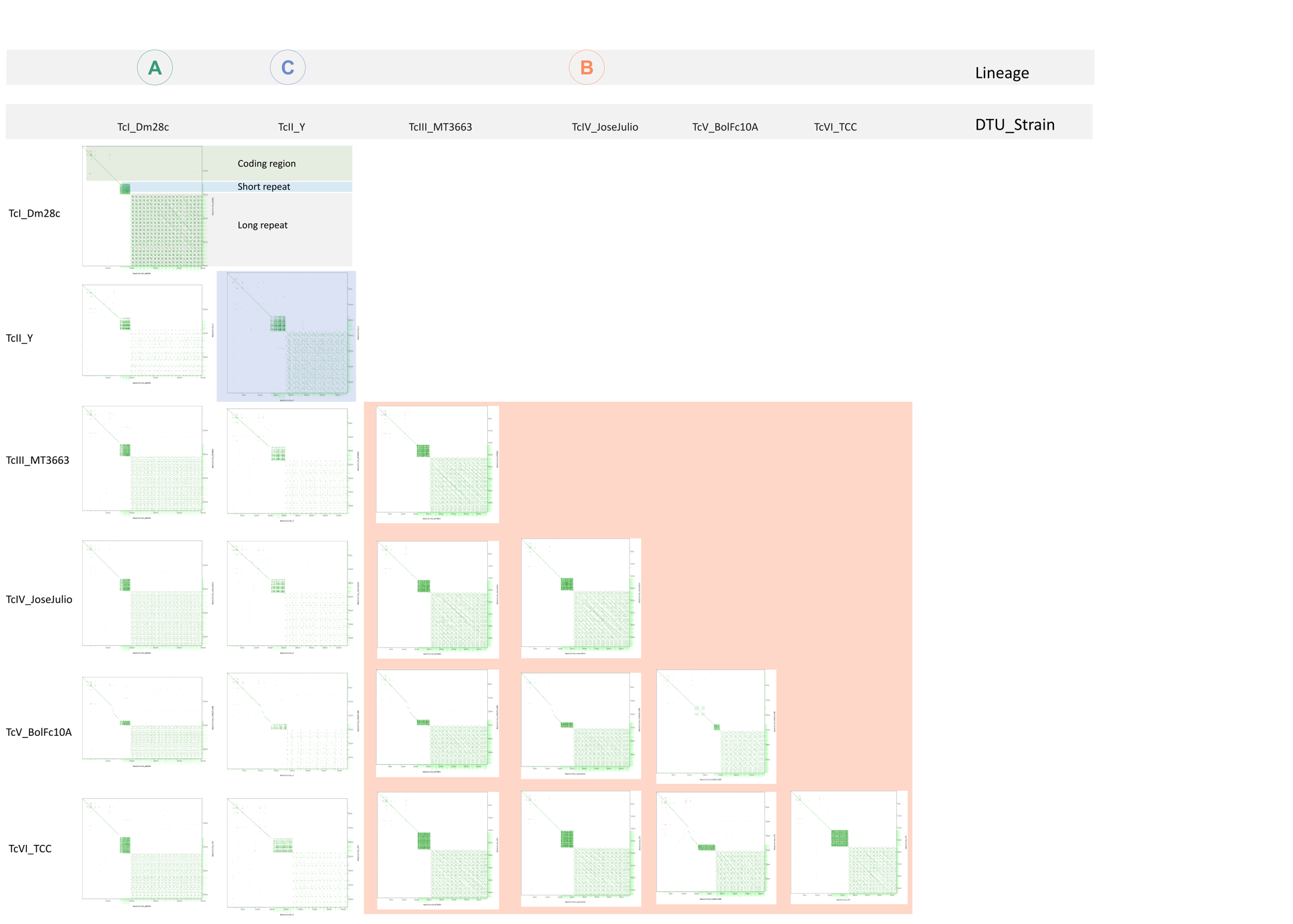

Supplement: S4 Fig — (TIF) [file pntd.0009719.s004.tif]

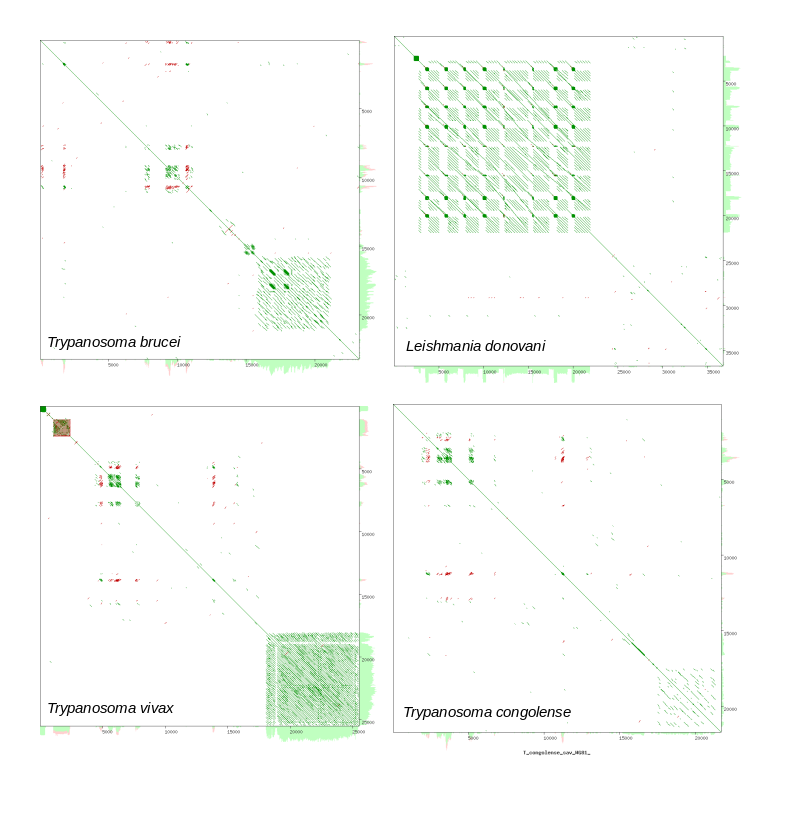

Supplement: S5 Fig — (TIF) [file pntd.0009719.s005.tif]

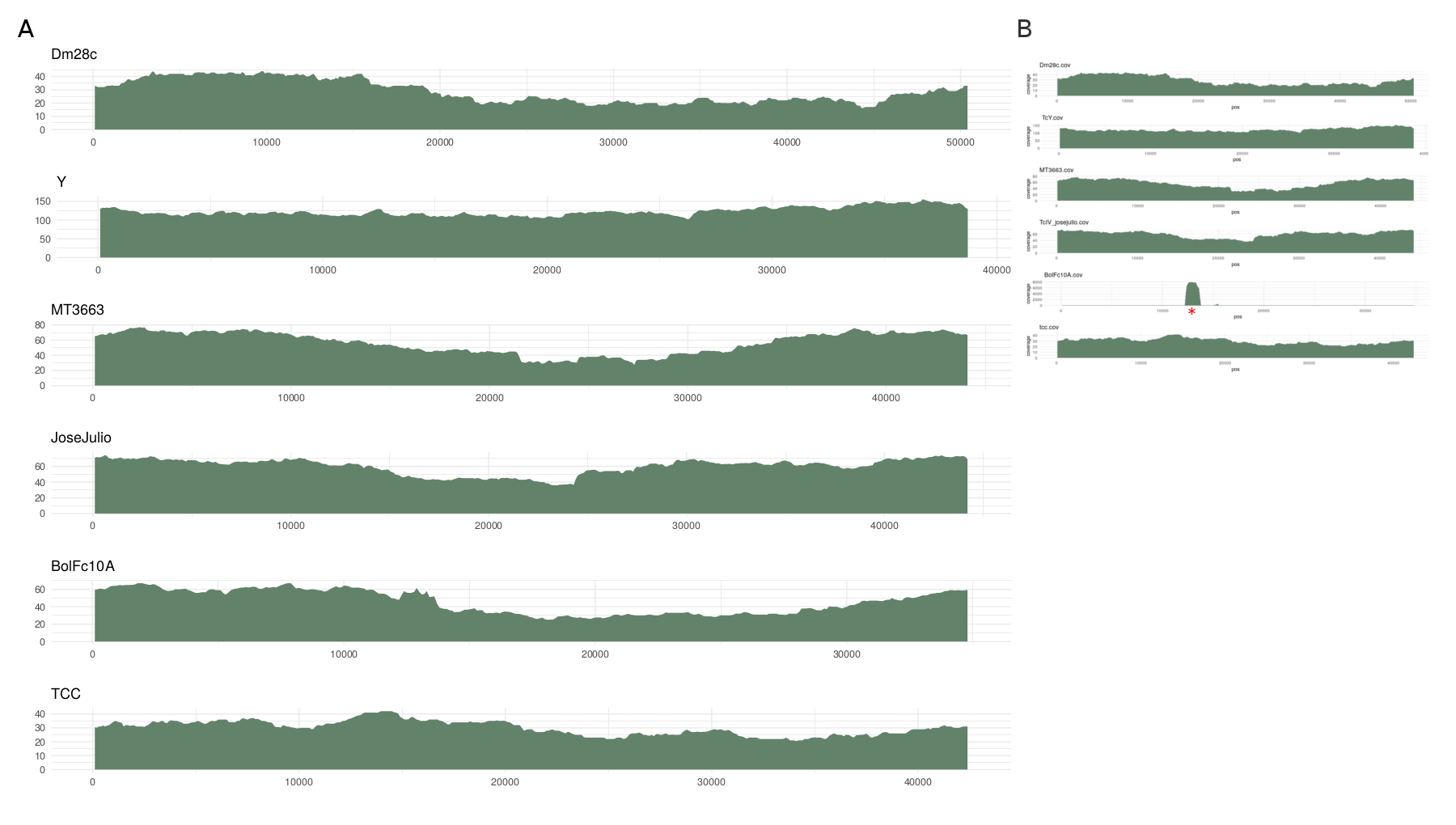

Supplement: S6 Fig — (TIF) [file pntd.0009719.s006.tif]

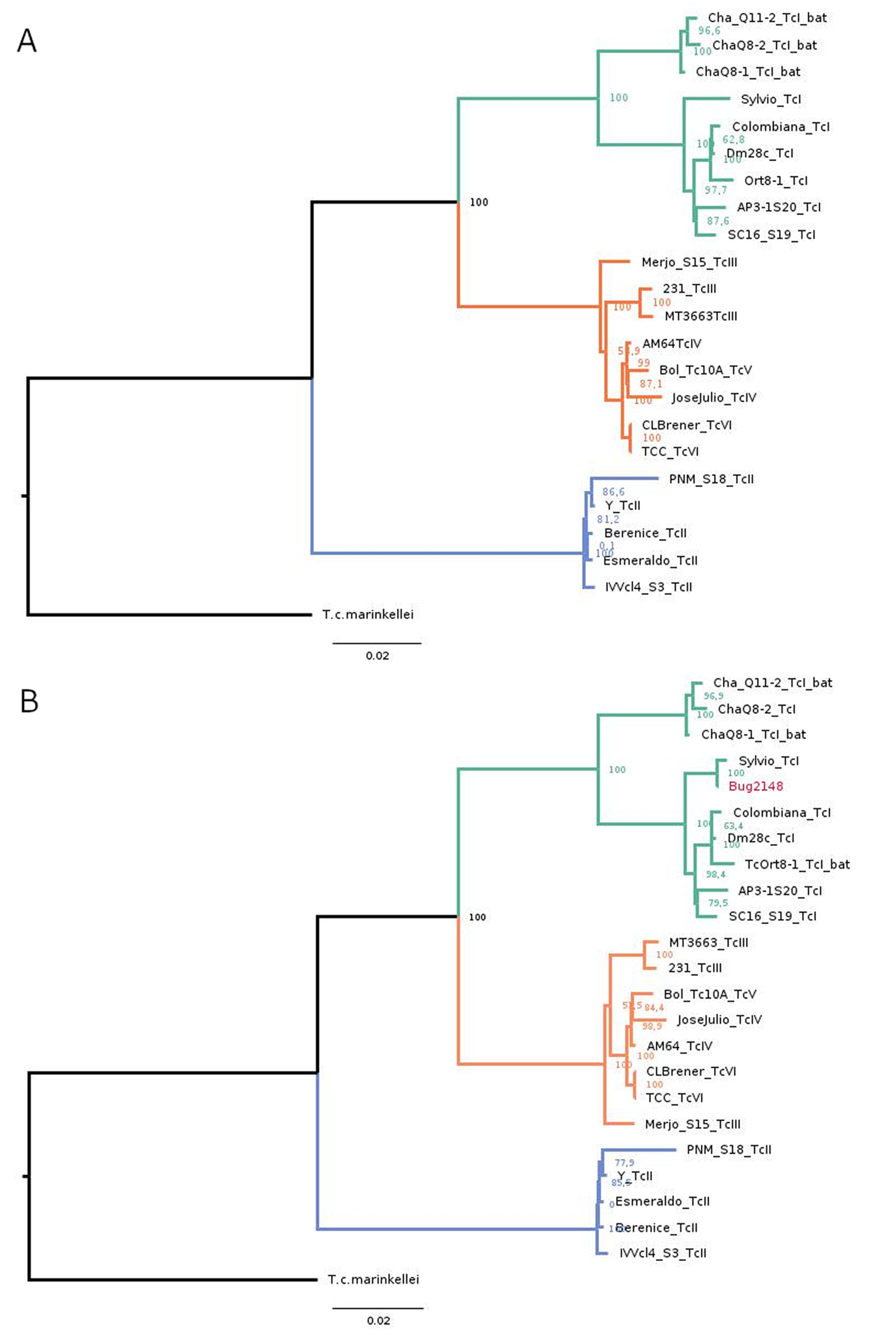

Supplement: S9 Fig — (TIFF) [file pntd.0009719.s009.tiff]

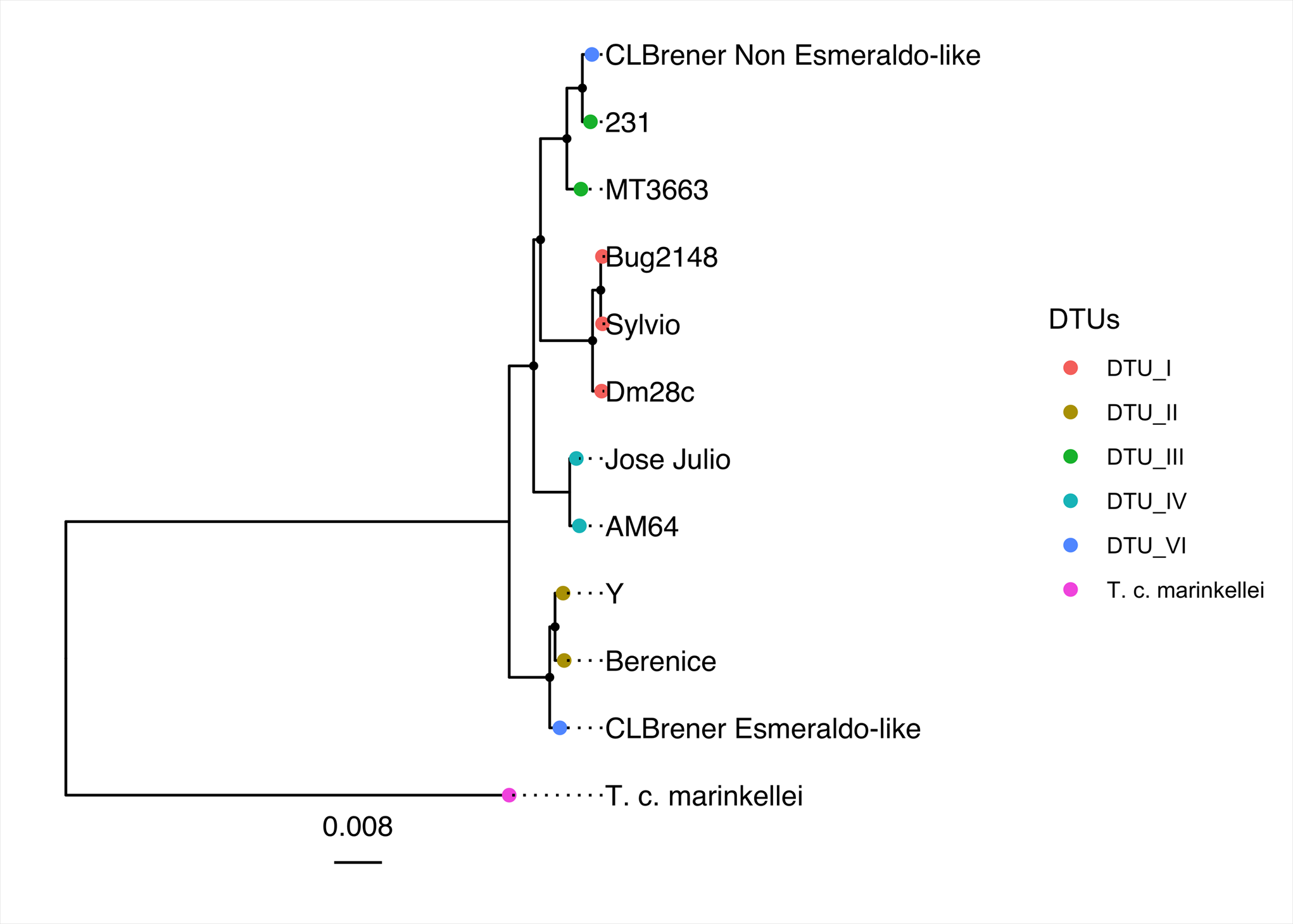

Supplement: S10 Fig — (TIFF) [file pntd.0009719.s010.tiff]
