# Supplementary material for: Maxicircle architecture and evolutionary insights into Trypanosoma cruzi complex
Source: PLoS Negl Trop Dis. 2021 Aug 26;15(8):e0009719. doi: 10.1371/journal.pntd.0009719 (PMC8425572; doi:10.1371/journal.pntd.0009719)
Supplement: S2 Table — (DOCX) [file pntd.0009719.s012.docx]

| S2 Table | | |
| --- | --- | --- |
| Strain | **DTU** | Technology |
| AP3-1 | TcI | Illumina |
| Colombiana | TcI | Illumina |
| SC16 | TcI | Illumina |
| Sylvio X10 cl1 | TcI | Illumina |
| Ort8-1 | TcI | Illumina |
| Cha_Q11-2 | TcI (Bat) | Illumina |
| ChaQ8-2 | TcI (Bat) | Illumina |
| ChaQ8-1 | TcI (Bat) | Illumina |
| Y | TcII | Illumina + ONT |
| PNM | TcII | Illumina |
| Berenice | TcII | Illumina |
| Esmeraldo cl3 | TcII | Illumina |
| IVV cl4 | TcII | Illumina |
| MT3663 | TcIII | Illumina + ONT |
| Merjo do Anjico | TcIII | Illumina |
| 231 | TcIII | Illumina |
