# Supplementary material for: Maxicircle architecture and evolutionary insights into Trypanosoma cruzi complex
Source: PLoS Negl Trop Dis. 2021 Aug 26;15(8):e0009719. doi: 10.1371/journal.pntd.0009719 (PMC8425572; doi:10.1371/journal.pntd.0009719)
Supplement: S1 Data — (DOCX) [file pntd.0009719.s013.docx]

**S1 Data**

C4B63_23g188, C4B63_1g55, C4B63_2g46, C4B63_2g52, C4B63_2g770, C4B63_2g768, C4B63_3g1103, C4B63_4g91, C4B63_4g360, C4B63_4g350, C4B63_4g182, C4B63_4g256, C4B63_6g227, C4B63_6g230, C4B63_9g522, C4B63_9g494, C4B63_9g434, C4B63_9g326, C4B63_9g315, C4B63_9g253, C4B63_9g243, C4B63_9g239, C4B63_9g1532c, C4B63_9g228, C4B63_10g406, C4B63_10g136, C4B63_11g93, C4B63_12g399, C4B63_12g298, C4B63_13g37, C4B63_16g81, C4B63_17g120, C4B63_18g69, C4B63_18g78, C4B63_18g213, C4B63_18g211, C4B63_18g1035c, C4B63_21g279, C4B63_24g318, C4B63_25g308, C4B63_27g196, C4B63_28g29, C4B63_28g38, C4B63_30g237, C4B63_32g220, C4B63_35g591c, C4B63_38g57, C4B63_38g60, C4B63_38g61, C4B63_41g81, C4B63_42g254, C4B63_42g252, C4B63_42g229, C4B63_45g225, C4B63_49g112, C4B63_51g197, C4B63_52g164, C4B63_54g172, C4B63_54g13, C4B63_55g173, C4B63_55g60, C4B63_55g65, C4B63_58g47, C4B63_59g129, C4B63_59g130, C4B63_59g132, C4B63_59g134, C4B63_70g87, C4B63_70g77, C4B63_70g68, C4B63_70g67, C4B63_70g61, C4B63_75g70, C4B63_85g86, C4B63_85g85, C4B63_87g38, C4B63_93g79, C4B63_97g22, C4B63_97g25, C4B63_101g21, C4B63_101g35, C4B63_112g67, C4B63_112g66, C4B63_127g32, C4B63_149g26
