# Supplementary material for: Maxicircle architecture and evolutionary insights into Trypanosoma cruzi complex
Source: PLoS Negl Trop Dis. 2021 Aug 26;15(8):e0009719. doi: 10.1371/journal.pntd.0009719 (PMC8425572; doi:10.1371/journal.pntd.0009719)
Supplement: Attachment — Submitted filename: Response to reviewers PLOSNTD.docx [file pntd.0009719.s015.docx]

**Response to reviewers PLOSNTD**

**Reviewer's Responses to Questions**

Reviewer #1: see below

Reviewer #2: the objectives are clear and the methodology adopted is adequate

Reviewer #3: Very clear explanation of the purpose of the study and approaches adopted.

**We thanks to the reviewers their comments.**

--------------------

**Results**

-Does the analysis presented match the analysis plan?</br>

-Are the results clearly and completely presented?</br>

-Are the figures (Tables, Images) of sufficient quality for clarity?</br></br>

Reviewer #1: see below

Reviewer #2: the results are presented in well-structured figures that adequately show the data obtained.

Reviewer #3: Very nice main figures, supported by comprehensive supplementary data.

**We thanks to the reviewers their comments.**

--------------------

**Conclusions**

-Are the conclusions supported by the data presented?</br>

-Are the limitations of analysis clearly described?</br>

-Do the authors discuss how these data can be helpful to advance our understanding of the topic under study?</br>

-Is public health relevance addressed?</br></br>

Reviewer #1: see below

Reviewer #2: I believe the discussion is the article's weakness. The data obtained do not contradict the data available in the literature about the nomenclature of T. cruzi strains. There are also extrapolations about hybridization events that are not adequately supported by the data. Other problems and suggestions are presented in the Summary and General Comments section

**Taking into account the reviewer's comments, in which we agree, we deleted from the abstract the sentence "No significant differences were found in hybrid strains that justify the existence of TcV and Tc VI as separate clades: our results indicate that a unique event of hybridization between TcII and TcIII occurred" (lines 34-36), and from the Discussion we removed the sentence "A very relevant point is the case of hybrid strains. Our results indicate that a unique event of hybridization occurred in T. cruzi and hence, TcV and TcVI cannot be separated at the genomic level, and this was the reason by which we grouped them as BCb. Analysis of a greater number of genomes will allow confirming this result." (lines 353-357).**

Reviewer #3: Very nice discussion, which accurately and fairly summarises the new data and provides context for the findings.

**We thanks to the reviewer their comments.**

--------------------

Editorial and Data Presentation Modifications?

Use this section for editorial suggestions as well as relatively minor modifications of existing data that would enhance clarity. If the only modifications needed are minor and/or editorial, you may wish to recommend “Minor Revision” or “Accept”.

Reviewer #1: see below

Reviewer #2: in figure 5 the TcV strain is incorrectly named as TcVI in nuclear phylogeny.

**We thank the reviewer for the observation. Figure 5 was now corrected.**

Reviewer #3: No changes needed.

--------------------

**Summary and General Comments**

**Reviewer #1:** The authors present a very nicely executed study of T. cruzi maxicircle sequence assembly and diversity alongside a more limited analysis of nuclear sequences. The long read sequencing, corrected via illumine short reads, offers the most complete insight of maxicircle structure yet attempted. Furthermore, key findings – a large coding indel in the ND5 gene in two strains, and a apparently complete (or near complete) minicircle encoded in the maxicircle sequence – pose exiting questions, the former in relation to phenotypic differences between strains and the latter maybe shedding light on the origin minicircles themselves.

**We appreciate the considerations regarding this study and the results found, and we appreciate the reviewer's comments.**

I must say I found the other focus of the paper, a re-exploration of T. cruzi intraspecific nomenclature, somewhat less convincing – in fact this element rather dilutes the interesting findings above. I was party to the discussion around the 2012 re-assigning of T. cruzi nomenclature. The resultant TcI – TcVI lineage assignment was as much about politics of the time as it was about biology. Such is the nature of compromise. I’m not sure how useful re-visiting the debate might be. That said, I do not object to the inclusion on the debate in the paper.

**Thank you for your comments. We agree with the reviewer that the DTU definition constitutes a very useful tool in the context of defining a common language. Our main concern is with respect to recent posts where more DTUs are proposed. If we consider that the main clades are four, then that will help to reduce the confusion regarding the complex scenario shown by T. cruzi. In our opinion both nomenclatures complement each other.**

I do, however, worry about the claim that TcV and TcVI are the progeny of the same cross and/or identical. If you look at multiple TcV and TcVI strains, fixed genetic differences between these clades, and fixed similarities within point clearly to separate origins (see /10.1371/journal.pntd.0001363 for example). As such I suggest the authors reserve judgement on this until multiple strains from each (V/VI) have been sequenced.

**We fully agree with the reviewer. In this revised version, from the abstract we deleted the sentence "No significant differences were found in hybrid strains that justify the existence of TcV and Tc VI as separate clades: our results indicate that a unique event of hybridization between TcII and TcIII occurred" (lines 34-36), and from Discussion we removed the sentence "A very relevant point is the case of hybrid strains. Our results indicate that a unique event of hybridization occurred in T. cruzi and hence, TcV and TcVI cannot be separated at the genomic level, and this was the reason by which we grouped them as BCb. Analysis of a greater number of genomes will allow confirming this result." (lines 353-357).**

I also think the authors should be careful about dismissing the possibility if maxicircle ingression without nuclear mixis – the authors present no data to the contrary. The clearest instances are the introgression of TcIV (b) maxicircles into TcI (e.g. PMC3323513) where there is no evidence of hybrid nuclear genomes split between divergent nuclear clades.

**We agree with the reviewer, and that is the reason by which we wrote "Finally, to shed light on the origin of the close relationship between B and D, including the fact that they share the same maxicircle, whole genome analysis will help determine how hybridization and/or introgression events have occurred." in lines 357-359.**

Overall the study is well written, with some checking of English necessary. The tone is occasionally a little pugnacious – but there is no harm in that I suppose.

**Thank you for your comment. We have cheeked English and corrections were introduced. Concerning the tone, it was not our intention to be pugnacious. We hope that with the removal of some sentences the tone has changed.**

Minor comments

Numbered lines would be very helpful in future.

**Done**

Typo ‘Tse-Tse’ – Tsetse

**Done (line 59)**

Introduction – the sentences on vectors on the bottom of page three needs re-drafting – they are poorly wriiten

**Done (lines 59-60)**

Good use of the term 'dark matter'

**We now substituted the term for "resolution" (line 86)**

Results

Can you clarify what is going on with Bug2148 - this seems to be a labeling error / laboratory mix up ?

**We have modified this paragraph to make it more clear (lines 166-173. We do not know if they mixed the strains or there is a labeling error. In any case we have already contacted them and tell about our findings, so we suppose/hope they will solve this mistake through an erratum in the publication and in the TriTrypdb.**

Did you detect any evidence for variation among maxicircles from the same clone

**We specifically looked for that, and did not find variation on a same clone.**

Surprised by how few single copy genes you could find orthologues between the Tc genomes - 85 unique conserved ?

**We just found 85 single copy genes when all of the strains were used. If we exclude the hybrid strains, then 1023 single copy genes are detected (probably due to the hybrid nature of those strains). We now modified the text since it was not clear (lines 182-184)**

Top of page 11 - do you mean 40 KDa or 40 Kbp ? Also - could you change the wording of 'To those authors our recognition.' – sounds a bit odd.

Again - please be careful of sounding too dismissive - 'overvaluation of modern techniques' a little strong. Perhaps shift focus to emphasis the validity of more traditional approaches 'irruption' – is not a word

**We agree with the suggestions, and in fact they are 40 Kbp (and not KDa) We now modified the paragraph (lines 207-214)**

Check where the acronyms CR and SR appear - they are somewhat confusing – make sure the appear where they are first used

**We have checked them, used first in line 25.**

‘Westemberg’ typo – is it not Westenberger ?

**Yes. We corrected it (Line 270)**

**Reviewer #2:** The manuscript is very important due to the assembly of the mitochondrial genome of different strains of T. cruzi. The results are interesting and the description of the structure of the mitochondrial genome in four regions is one of the most important data in the article.

**We appreciate the considerations regarding this study and the results found, and we thank the reviewer for their comments.**

I do not agree with the final part of the discussion of the article, because all data presented agree with the separation of T. cruzi strains into DTUs-I to VI. The article does not present any evidence that only one hybridization event occurred, and the phylogenetic tree presented suggests two hybridization events by the position in the tree of the TcV and TcVI strains.

**In agreement with the reviewer, in this new version we have relativized this observation, stating more strongly that hybridization should be studied in more detail through the sequencing of new genomes.**

It would be much more relevant for the manuscript to argue that the data obtained support the hypothesis of 4 ancestors, and it is contrary to the hypothesis that TCIII is the result of the hybridization event between TCI and TCII.

Furthermore, it would be interesting to discuss why type b mitochondria prevails in all hybridization events and it is present in TcIV, can the size of the AT region favor the replication of this type of mitochondria?

**In view of these reviewer's comments, we have modified the manuscript, stating that both classifications are not exclusive (TcI-VI and A to D)and highlighting the observation of four ancestors.**

Reviewer #3: In this manuscript, Berná and colleagues apply long-read, mainly Nanopore, DNA sequencing to examine the sequence composition of one of the two circles (the maxicircle) that make up the highly unusual, catenated DNA network of the Trypanosoma cruzi mitochondrial genome, which is called the kinetoplast. To do so, the authors compare maxicircle sequence from a number of parasite strains that span six of the discrete typing units (DTUs) of the T. cruzi species complex. Using these data, they reveal two aspects of T. cruzi biology. First, they reveal that most of the maxicircle genome is composed of 3 different types of repetitive sequence; thus, they reveal the ‘dark matter’ within the genome. Second, they use the newly assembled genomes of the DTUs to revisit the classification of strains and clades within the T. cruzi complex, and suggest a simplification is needed.

In the interests of full disclosure, I confess that I was asked to review an earlier version of this paper, which was submitted to another journal, where it was ultimately rejected due to two main issues: lack of information around the potential functions of the new sequence elements revealed by long-read sequencing; and an over-assertive demand that T. cruzi DTU conventions be overturned by this work. This updated version of the paper is much improved, and both of these concerns have been admirably and comprehensively dealt with.

As such, I consider this submission to be very valuable: the sequencing efforts and results are comprehensive and will provide a rich data source for future work, and I now feel that the paper could initiate a useful discussion about the nomenclature used in the T. cruzi complex (indeed, the discussion is very fair and balanced in this regard). On this basis, I am content that the paper be accepted without (further) revision.

**We thanks the reviewer for her/his considerations and comments.**

--------------------
